# Supplementary material for: Association between tocilizumab, sarilumab and all-cause mortality at 28 days in hospitalised patients with COVID-19: A network meta-analysis
Source: PLoS One. 2022 Jul 8;17(7):e0270668. doi: 10.1371/journal.pone.0270668 (PMC9269978; doi:10.1371/journal.pone.0270668)
Supplement: S1 Table — (DOCX) [file pone.0270668.s002.docx]

**Supplemental Table 1:** Certainty assessment for each comparison

| **Comparison** | **Direct estimate** | | | | | **Indirect estimate** | | | **Net estimate** | | | |
| --- | --- | --- | --- | --- | --- | --- | --- | --- | --- | --- | --- | --- |
|  | ***Risk of Bias*** | ***Inconsistency*** | ***Indirectness*** | ***Publication bias*** | ***Rating*** | ***Lowest rating in dominant loop*** | ***Intransitivity*** | ***Rating*** | ***Rating of dominant estimate*** | ***Incoherence*** | ***Imprecision*** | ***Final rating*** |
| Tocilizumab vs usual care or placebo | Not serious | Not serious | Not serious | Not serious | High | - | - | - | High (direct) | Not serious | Not serious | High |
| Sarilumab vs usual care or placebo | Serious | Not serious | Not serious | Not serious | Moderate | High | Not serious | High | High (indirect) | Not serious | Serious | Moderate |
| Tocilizumab vs sarilumab | Not serious | Not serious | Not serious | Not serious | High | - | - | - | High (direct) | Not serious | Serious | Moderate |

Tocilizumab vs usual care or placebo and tocilizumab vs sarilumab do not require rating of indirect evidence as the direct evidence contributes more than the indirect evidence for the net estimate in these comparisons and the direct estimate for both is rated as High
