# Supplementary material for: Association between tocilizumab, sarilumab and all-cause mortality at 28 days in hospitalised patients with COVID-19: A network meta-analysis
Source: PLoS One. 2022 Jul 8;17(7):e0270668. doi: 10.1371/journal.pone.0270668 (PMC9269978; doi:10.1371/journal.pone.0270668)
Supplement: Attachment — Submitted filename: IL6 NMA response to reviewers_v3.docx [file pone.0270668.s003.docx]

**Association between tocilizumab, sarilumab and all-cause mortality at 28 days in hospitalised patients with COVID-19: A network meta-analysis: Response to reviewers document**

| No. | Comment | Response |
| --- | --- | --- |
| REVIEWER 1 | | |
| - | Thank you for the opportunity to review this rigorous and well-conducted network meta-analysis. I have only a few minor points for the authors to consider to enhance the interpretation of the study. One of the major rationales for addressing the question of tocilizumab vs. sarilumab is that clinicians tend to favour the former. The study partially addresses this question, but a few issues remain. | Thank you for your positive comments and helpful suggestions. We have taken these on board in our revised manuscript. |
| 1 | No evidence for this claim (page 3, lines 71-72) "Possibly related to interpretations of these findings, there is some emerging evidence to suggest that clinicians and healthcare providers have tended to favour the use of tocilizumab, with potential implications on patient access to these treatments." Anecdotally, this statements aligns with my experience but I am not sure whether it is true or not, and no citation is provided. | Thanks for this point, we do not have any formal citable reference to support this point, and are only aware of this anecdotally, for example, we were asked to supply an early draft of the results presented in this manuscript to Public Health England after they enquired about using Sarilumab once Tocilizumab stores had diminished.  Therefore, we have changed the wording to make it clear that this statement is based on anecdotal evidence rather than formal citable evidence. See changed text in the Introduction (page 3, Lines 71-73):  *“If, based on these findings, clinicians and healthcare providers tend to favour the use of tocilizumab, there will inevitably be implications on demand and availability, potentially limiting patient access to tocilizumab.”* |
| 2 | There was no consideration of the quality/certainty of evidence across studies, eg. GRADE. This is quite feasible to do even in the NMA setting (https://www.bmj.com/content/349/bmj.g5630). Consideration of imprecision, inconsistency, indirectness, incoherence, etc. in a structured way (I would advocate for GRADE) could provide the reader with a better sense of how confident we should be in these results. For instance the confidence intervals for the T vs. S comparisons are very wide in all 3 comparisons (direct, indirect, net); the potential for incoherence between direct vs. indirect T vs. S comparisons, etc. This is not merely a matter of academic interest, but also important in exploring the study question regarding why clinicians may favour T vs. S (eg. direct evidence is less precise, etc.) | We have now undertaken and included a GRADE assessment to appraise the certainty of the evidence, as suggested. This has resulted in additional text in the methods, results, a new supplementary table (S1 Table) and additional text in the discussion.  Added text in the Methods (page 6, Lines 155-158):  *“The certainty of evidence in each comparison was determined following the GRADE approach to network meta-analysis[14, 15], with this completed independently by two reviewers [PJG and CLV] and any discrepancies resolved through discussion. The certainty of evidence was rated as either High, Moderate, Low or Very Low.”*  Here reference 15 is the reference suggested by Reviewer 1 and reference 14 is one of the references suggested by Reviewer 2.  Added text in the Results (Page 8, Lines 214-216):  *“The certainty of evidence for each comparison is displayed in S1 Table. Tocilizumab versus usual care was rated as High, with sarilumab versus usual care and tocilizumab versus sarilumab both rated as Moderate (both downgraded due to Imprecision).”*  Added text in the Discussion (Page 9, Lines 241-244):  *“The authors also down-rated the certainty of evidence for tocilizumab versus usual care, sarilumab versus usual care and tocilizumab versus sarilumab, as Moderate, Low and Low respectively, based on a lack of blinding in many of the included trials.”*  And further added text in the Discussion (Page 9, Lines 253-258):  *“Furthermore, given that over 80% of the total events included in this network were from trials judged to be at low risk of bias, and because of a lack of subjectivity in the assessment of a mortality outcome, we have kept our risk of bias assessments consistent with the approach used in the prospective meta-analysis[1] and by the WHO guideline panel[2]. Consequently, we rated the certainty of evidence as High, Moderate and Moderate for the comparisons of tocilizumab versus usual care, sarilumab versus usual care and tocilizumab versus sarilumab, respectively.”* |
| 3 | Lastly, the discussion does little to address the question which the NMA claims to address, re: clinician practice. While making such recommendations is more likely to be done in the setting of a guideline, it is important to note that the rationale for clinicians and patients choosing a treatment is based upon many factors (desirable effects; undesirable effects for sure; but also certainty of those effects, the balance, the feasibility, cost, acceptability etc.). While the review does not really address all these other issues, the discussion could note that these are also important, not just the mortality rate alone (as important as that is). These are fairly minor concerns in a wonderful paper. Butt as it quite explicitly asks one question, I expected there to be greater exploration of the certainty of the effects of T vs. S and some discussion on the topic (including all the various factors for choosing a treatment which are not really addressed in this NMA). | A full exploration of both treatments (tocilizumab and sarilumab) compared with placebo or standard care on a full range of outcomes, and assessed within and across patient subgroups was presented in the prior prospective meta-analysis paper (ref 1 of manuscript).  The purpose of this network meta-analysis was to complete the circle, by incorporating data from direct comparisons of the two agents – which is limited to that from the REMAP-CAP trial, and to provide an assessment of their relative effects on the primary outcome from the prior meta-analysis.  We agree with the reviewer that the most likely way for this results to influence practice is through clinical guidelines which will require an assessment of the certainty of the effects. We have now included the GRADE assessment, as described above.  We did not set out, nor do we have the data to support any formal recommendation/guideline not to comment on when one treatment might be used over another. This work has been conducted in close collaboration with the WHO, which has a process in place for developing guidelines on the optimal treatment of COVID-19, and we defer to the WHO in making specific clinical recommendations. |
| REVIEWER 2 | | |
| 1 | The authors did not assess the certainty of the evidence, which- according to Cochrane- is considered a methodological standard for all systematic reviews. This can be done using GRADE or CINEMA They should not only add this assessment (which includes specifying the degree of contextualization they will use and their target of certainty), but incorporate it formally in the drawing of their conclusions regarding the effects of the interventions. They can find detailed information about certainty of evidence assessment process and drawing conclusions from NMA in the GRADE for NMA papers, making narrative statements/ conclusions in GRADE guidelines 26, and contextualization/ target of certainty rating in GRADE guidelines 32. | Thank you for this suggestion, we have now added a GRADE assessment. Please see our response to reviewer 1, point 2. |
| 2 | In addition, authors should follow the prisma statement for NMA for reporting. | Thank you for this suggestion. We had reported according to PRISMA-NMA, but have now added some text in the methods to make this explicit. Note that our checklist is included in the supplemental material.  See added text in Methods (page 4 lines 99-100):  *“This network meta-analysis is reporting according to the Preferred Reporting Items for Systematic Reviews and Meta-Analyses extension statement to Network Meta-Analyses [6] (see S1 Checklist).”*  Here, reference 6 is the PRISMA extension to NMA. |
